# Life Stressors, Social Capital, and Mental Distress in Multiethnic Groups of Older Asian Americans

**DOI:** 10.1093/geroni/igaa057.2171

**Published:** 2020-12-16

**Authors:** Yong Ju Cho, Audrey Hai, Yuri Jang

**Affiliations:** 1 University of Southern California, Los Angeles, California, United States; 2 University of Texas at Austin, Austin, Texas, United States

## Abstract

Using data from older participants from the 2015 Asian American Quality of Life survey (n = 533), the present study assessed direct and interactive effects of life stressors and social capital. The sample includes diverse Asian ethnic groups (Chinese, Asian Indian, Korean, Vietnamese, and others). Among all groups, high levels of mental distress were found in Koreans and Vietnamese. In the multivariate analyses, Korean ethnicity (compared to Chinese) was found to be a significant predictor to mental distress. As significant risk factors to mental distress, all stressor variables accounted for 9% of the variance of mental distress. Social capital variables explained the variance by 4%. None of the interaction terms reached statistical significance. Findings confirmed the negative effects of stressors and the positive effects of social resources across older Asian Americans. However, it was interesting to note that ethnic variations disappeared when stressors and social capitals were taken into considerations.

